# Team Sport in the Workplace? A RE-AIM Process Evaluation of ‘Changing the Game’

**DOI:** 10.3934/publichealth.2017.5.466

**Published:** 2017-10-31

**Authors:** Andrew Brinkley, Hilary McDermott, Fehmidah Munir

**Affiliations:** School of Sport, Exercise and Health Sciences, National Centre for Sport and Exercise Medicine, Loughborough University, Loughborough, Leicestershire LE11 3TU, UK

**Keywords:** acceptability, effectiveness, feasibility, health, intervention, workplace

## Abstract

**Background:**

The workplace is a priority setting to promote health. Team sports can be an effective way to promote both physical and social health. This study evaluated the potential enablers and barriers for outcomes of a workplace team sports intervention programme ‘Changing the Game’ (CTG). This study was conducted in a FTSE 100 services organisation. This process evaluation was conducted using the RE-AIM framework.

**Methods:**

A mixed methods approach was used. Data were collected from the participants in the intervention group prior to, during and at the end of the intervention using interviews (*n* = 12), a focus group (*n* = 5), and questionnaires (*n* = 17). Organisational documentation was collected, and a research diary was recorded by the lead author. The evidence collected was triangulated to examine the *reach, efficacy, adoption, implementation and maintenance* of the programme. Data was assessed through template analysis, and questionnaire data were analysed using multiple regression and a series of univariate ANOVAs.

**Results:**

CTG improved VO_2_ Max, interpersonal communication, and physical activity behaviour (efficacy) over 12-weeks. This may be attributed to the supportive approach adopted within the design and delivery of the programme (implementation). Individual and organisational factors challenged the adoption and maintenance of the intervention. The recruitment and communication strategy limited the number of employees the programme could reach.

**Conclusion:**

The process evaluation suggests addressing the culture within workplaces may better support the reach, adoption and maintenance of workplace team sport programmes. Future research should consider investigating and applying these findings across a range of industries and sectors.

## 1. Introduction

Globally, physical activity guidelines are failing to be met [1]. Inactivity predicts non-communicable illnesses and premature mortality [2]. An inactive workplace has a greater prevalence of absenteeism and presenteeism, occupational fatigue, reduced productivity and work-engagement [3],[4]. Per-annum $67.5 billion is estimated to be spent on the global direct (i.e., health-care) and indirect (i.e., productivity) cost of inactivity [3].

Due the time employees spend in an organisational setting and the stability of this environment, the workplace presents an ideal setting to promote health through participation in modes of physical activity [4]. Employers are thought to promote participation in physical activity due to social responsibility, improved productivity, reduced sickness absence and a duty of care [4]. However, little is known about how individual modes of physical activity such as sport are adopted by organisations [4],[5]. A robust process evaluation understands the factors contributing to uptake, acceptability, feasibility and efficacy of a given mode of workplace physical activity [6].

Workplace team sport is becoming a popular means to promote employee and organisational health [5]. However, evidence investigating workplace team sport is limited by a lack of validated instruments and poor descriptions of team sport [5]. Further, the evidence evaluating workplace team sport is yet to conduct process evaluations in accordance with the guidelines of the Medical Research Council [6]. Omitting information on uptake, adherence and feasibility limits the case for employers considering offering team sport to their employees.

Whilst interventions studies are useful in exerting changes in outcomes measures, they do not provide clarity in how changes in outcomes may have occurred or how outcomes may have been influenced by individual and organisational factors [6],[7]. Moreover, without a robust process evaluation, little is known as to whether the intervention is acceptable or feasible, or indeed translatable to a real-world setting [6],[7].

One method to evaluate workplace team sport is through the RE-AIM framework [7]. The RE-AIM framework translates evidence into real-world applications, through investigating efficacy, feasibility and acceptability of an intervention [7]. This framework explores the individual, organisational, environmental factors and policies which influence the *reach, efficacy, adoption, implementation* and *maintenance* of an intervention study [7]. *Reach* can be considered the total number of individuals available to take part in a study; and the proportion of, and characteristics of individuals willing to participate [7]. *Efficacy* is the impact of the study on key outcomes (e.g., health, psychological wellbeing) [7]. In the case of the current study, the efficacy has been previously examined in Brinkley et al. [8]. This non-randomised design tested the effectiveness of providing a programme of multiple team sports within a workplace setting. *Adoption* refers to the number of individuals who engage in the intervention study either initially and/or across its duration [7]. The *implementation* of a study seeks to understand if the intervention was conducted in accordance with the planned protocol, and the reasons why this may or may not have been the case [7]. Finally, the *maintenance* of an intervention study can be considered as the degree to which the intervention has been adopted beyond the end of the study period or the potential to be adopted, within the routine, structure or practices of an organisation [7]. A robust RE-AIM evaluation seeks to comprehensively explore the causality and mechanisms underpinning each outcome [7]. The RE-AIM framework is widely adopted within workplace health promotion [7].

Therefore, a RE-AIM process evaluation was conducted alongside the intervention study. The purpose of the intervention study reported in Brinkley et al. [8], was to explore the efficacy of workplace team sport over time. In contrast, the purpose of this process-evaluation was to explore the acceptability and feasibility of participation, and the perceived efficacy of the programme. This was conducted using a range of quantitative and qualitative methods, as per the recommendations of the MRC [6]. Evaluating health promotion programmes through the triangulation of a range of data sources is considered a robust form of investigation [6],[7]. The aim of the current study was to evaluate the acceptability, impact and feasibility of the CTG programme using the RE-AIM framework [7].

## 2. Methods

### 2.1. Study context and ‘Changing the Game’ overview

A non-randomized study (quasi-experimental design) was conducted to investigate a needs-supportive workplace team sport intervention designed to promote improvements in individual, social group and organisational health outcomes. The intervention, named ‘Changing the Game’ (CTG), was a 12-week team sport programme available to employees of a UK based FTSE 100 services organisation. A detailed overview of its design and findings over T^0^-T^1^ (12-weeks) are available in Brinkley et al. [8]. The intervention study tested the effectiveness of participation in workplace team sport on markers of individual health (e.g., VO^2^ Max, wellbeing), PA behaviour, work team outcomes (e.g., cohesion, communication) and workplace outcomes (e.g., productivity, sickness, occupational fatigue). The organisation's workforce consisted of 5080 employees. Two regional worksites were selected to take part in the study of which one served as the intervention group and the other worksite acted as the control group (normal working conditions arms). The intervention group participated in weekly lunchtime sessions of Rounders (weeks 1 & 7), Netball (weeks 2 & 8), Basketball (weeks 3 & 9), Soccer (weeks 4 & 10), Cricket (weeks 5 & 11) and Handball (weeks 6 & 12). The research team selected sports which use transferable skills, and are adaptable. CTG was conducted in an indoor sports hall located 400 metres from the participating organisation. The sessions were led by two female workplace champions.

The intervention components of CTG were underpinned by Self-Determination Theory [9],[10], which suggests supporting individual's innate needs for autonomy (i.e., feeling free and fully volitional to engage in team sport), competence (i.e., feeling capable to complete a skill in team sport) and relatedness (i.e., feeling supported, understood and valued by a social group) promotes wellbeing and autonomous motivation [9],[10]. Supporting basic needs can be achieved through creating an autonomy (needs) supportive environment (e.g., encouraging and offering a choice to individuals to decide or adapt the rules of the sport, impart the benefits of team sport, and not impose perceptions and experiences upon them) [9],[10]. Supported basic needs are known to improve wellbeing and autonomous motivation [9],[10]; and autonomous motivation is associated with maintained participation over time [9],[10]. Workplace champions implemented the autonomy supportive environment. Autonomy was promoted when champions reinforced the benefits of participation, offered a choice, and provided ownership and control to participants [9],[10]. Competence was supported by providing sports which were novel, adaptable and used transferable skills [9],[10]. Relatedness was supported through peer led sessions, the support of colleagues and membership and belonging to a workplace social group. The primary outcome of the intervention was VO^2^ Max, and additional measures of individual, social-group and organisational health were taken at T^0^ (baseline) and at T^1^ (12-weeks) [8]. A brief overview of the programmes outcome measures is presented within the findings under the heading ‘efficacy’, whilst a detailed overview is available in Brinkley et al. [8].

### 2.2. Participants

A small number of participants involved in the main study took part in the process evaluation. Prior to the implementation of CTG, five participants took part in a focus group. Following the end of the intervention, ten participants from the intervention group took part in semi-structured interviews and five participants from the control group took part in a focus group. The two workplace champions who delivered the intervention were also interviewed at the end of the intervention. Finally, 17 participants from the intervention group completed a short process evaluation questionnaire Employees from a broad range of office based roles, positions of superiority and departments within the organisation were represented [8] An overview of participation in the process evaluation, and the RE-AIM dimension addressed is provided in Table 1. Characteristics of the overall sample is presented in Table 2.

**Table 1.** Details of process evaluation participation. Dimensions assessed based on participants.

*Dimensions*	*Pre-intervention focus group (n = 5)*	*Study outcome measures (T^0^-T^1^) (n = 27)*	*Process-evaluation questionnaire (intervention group) (T^1^) (n = 17)*	*Post-intervention interviews (intervention group) (n = 10)*	*Post-intervention interviews (workplace champions) (n = 2)*	*Post-intervention focus group (control group) (n = 5)*
*Reach*	**X**		**X**	**X**	**X**	**X**
*Adoption*	**X**		**X**	**X**	**X**	**X**
*Efficacy*		**X**	**X**	**X**	**X**	
*Implementation*			**X**	**X**	**X**	**X**
*Maintenance*	**X**		**X**	**X**	**X**	**X**

**Table 2.** Changing the Game participant demographics at baseline.

	*Group*				*Sig*

	Team Sport		Control		

	M	SD	M	SD	
*Age*	39.59	9.11	40.75	11.92	0.708
*Gender (Male = M, Female = F)*	M = 20, F = 8		M = 8, F = 12		0.030 *
*Body Mass Index (BMI) (T^0^)*	27.71	4.49	26.28	5.09	0.931
*Tenure (Months)*	119.77	123.01	139.35	162.11	0.640
*Average Working Hours*	38.74	7.15	34.65	4.96	0.034 *
*Average Number of Teams*	2	1.46	1.3	0.73	0.057
*Average Team Size*	9.29	6.68	11.45	9.11	0.355
*Number of Superiors*	18		10		0.064

Significant interactions indicated with * *P* < 0.05, ** *P* < 0.01, *** *P* < 0.001.

### 2.3. Qualitative data collection (reach, efficacy, implementation, maintenance)

Prior to implementing CTG, a focus group was conducted with five employees (*n* = 2 females) who were considering participating in CTG consented to participate. Current physical activity experiences, expectations and challenges in taking part in workplace physical activity and team sports were discussed (e.g., *‘what physical activity do you currently participate in?’, ‘how do you feel about participating in workplace team sport?’*). During the intervention, the lead author, who attended all the CTG team sports sessions as an observer, kept a paper-based diary to record contextual information relating to the efficacy (e.g., *did participants communicate; was there cohesion in the group?*) and implementation (e.g., *was the programme being implemented as planned by workplace champions; did participants report any barriers*) of the programme. Throughout the duration of the intervention, 31 diary entries were recorded. Entries were recorded following the completion of each intervention session, and reflections were recorded between intervention sessions. Following the completion of CTG, semi-structured interviews were conducted with ten participants (*n* = 2 females) from the intervention group and the two workplace champions who delivered the intervention. Reach (e.g., *‘how did you find out about CTG?’*), efficacy (e.g., *‘how did participation in CTG benefit you?’*), adoption (e.g., *‘what prevented you from attending the programme?’*), implementation (e.g., ‘did you like the sports you played and how they were delivered?’) and maintenance (e.g., *‘are you still participating in team sport now the programme has finished?’*) of CTG were discussed. In order to explore the organisational factors influencing the reach, adoption, implementation and maintenance of CTG across the workforce, a focus group, with five participants (*n* = 4 females) from the control group, was conducted following the completion of the intervention (e.g., *‘what might stop you attending a team sport programme in your workplace?’*). Data collection was conducted by the first author^1^. Interview and focus group schedules are available from the corresponding author.

### 2.4. Process Evaluation Questionnaire (reach, efficacy, adoption, implementation, maintenance)

A process evaluation questionnaire was designed to explore the reach, efficacy, adoption, implementation and maintenance of the intervention, and examine the impact of the intervention and its components of the theoretical underpinnings of CTG. Seven open-ended items explored the intervention participants' experiences (efficacy) and perceptions of the CTG intervention programme (e.g., *‘what motivated or enabled you to take part in the programme?’*) (implementation). Two validated questionnaires were used to examine the impact of CTG on the three psychological aspects outlined by the Self-Determination Theory (implementation). These are the innate needs for (a) autonomy, (b) competence and (c) relatedness that may influence team sport participation and adherence. A modified version of the Sport Climate Questionnaire short-form [11] was used to assess autonomy of support provided by workplace champions (six 7-point Likert scale items; *‘I felt my workplace champion provided me with choices and options’*). A modified version of the Basic Needs in Sport Scale [12] was used to measure perceptions of basic needs satisfaction (e.g., *‘I can overcome challenges in the sports I played’*). Perceptions of competence (five items; *‘I was skilled at the sports I played’*), choice (four items; *‘In the sports I played, I had a say in how things were done’*), internal perceptions of the locus of causality (three items; *‘In the sports I played, I felt I was perusing goals that were my own’*), volition (three items; *‘I felt I participated in the sports willingly’*) and relatedness (five items; *‘I had a close relationship with the people I played sport with’*) are assessed with twenty 7-point Likert scale items. Wellbeing was assessed using the Subjective Vitality Scale (e.g., *‘At this moment I feel alive and vital’*) [13].

### 2.5. Documentation (reach, adoption, implementation, maintenance)

Reach was determined by the number of employees employed at the intervention and control worksite who expressed an interest in participating in the study during the recruitment phase of CTG. Adoption of the intervention programme was assessed by an attendance register recorded at each intervention session. Publicly available, annual reports (2013–2016) and governance documents from the participating organisation were collected to clarify the information provided by participants.

### 2.6. Data analysis

#### 2.6.1. Quantitative data

Data were assessed with IBM SPSS Statistics version 23, and *P* < 0.05 was considered statistical significant. Missing data were treated with within-person mean substitutions of the missing value. Self-reported measures were standardized to a 0–100 score (0 is considered unfavourable). Descriptive statistics were computed for all variables. Week by week sports session attendance was examined using a series of one-way ANOVAs. Standard linear multiple regressions examined if participation in an autonomy supportive team sport programme predicted changes in participants' basic needs and subjective wellbeing. Bivariate Pearson correlations were conducted on autonomy support and basic needs scores to examine if an autonomy supportive team sport programmes predicts autonomy, competence and relatedness. The assumptions associated with multiple regression were met for autonomy support and basic needs variables (no influencing multivariate outliers or leverage points, data met the assumptions for normality, homoscedasticity, linearity, multicollinearity). The data representing wellbeing did not have independence of observations and was removed from multivariate analysis.

#### 2.6.2. Qualitative data

Interviews and focus groups were transcribed verbatim. A template analysis [14], collectively incorporating data collected interviews, focus groups, questionnaire and diary data were undertaken using QSR International NVivo version 11. Template analysis is a form of thematic analysis which uses priori-themes to guide analysis towards a given research question, whilst allowing new themes to be identified and incorporated into the analysis [14]. Previous research has demonstrated template analysis to be a trustworthy and reflexive tool [14],[15]. Priori themes (e.g., reach, efficacy, adoption, implementation, maintenance, facilitators and obstacles to attendance, supporting basic needs and autonomy support) were based on previous research [5],[15],[16]. Familiarisation in the data created a series of codes. Codes were attached to priori themes where appropriate. Failure to attach a code, led to a new theme being developed. Once completed, a template was produced (available on request from first author). The template grouped data into first- (e.g., adoption), second- (e.g., obstacles to attendance) and third-level themes (interpersonal obstacles to participation). The template was revised, until it reflected the complete data set. All members of the research team gave their consensus on the data by reviewing the identified themes.

### 2.7. Ethical Approval

Ethical approval for this study was granted by Loughborough University's Human Participants Sub-Committee in April 2016 (see R16-P069). The study's outcome data has been published [8]. All participants provided written informed consent.

### 2.8. RE-AIM dimensional rating

**Figure 1.**
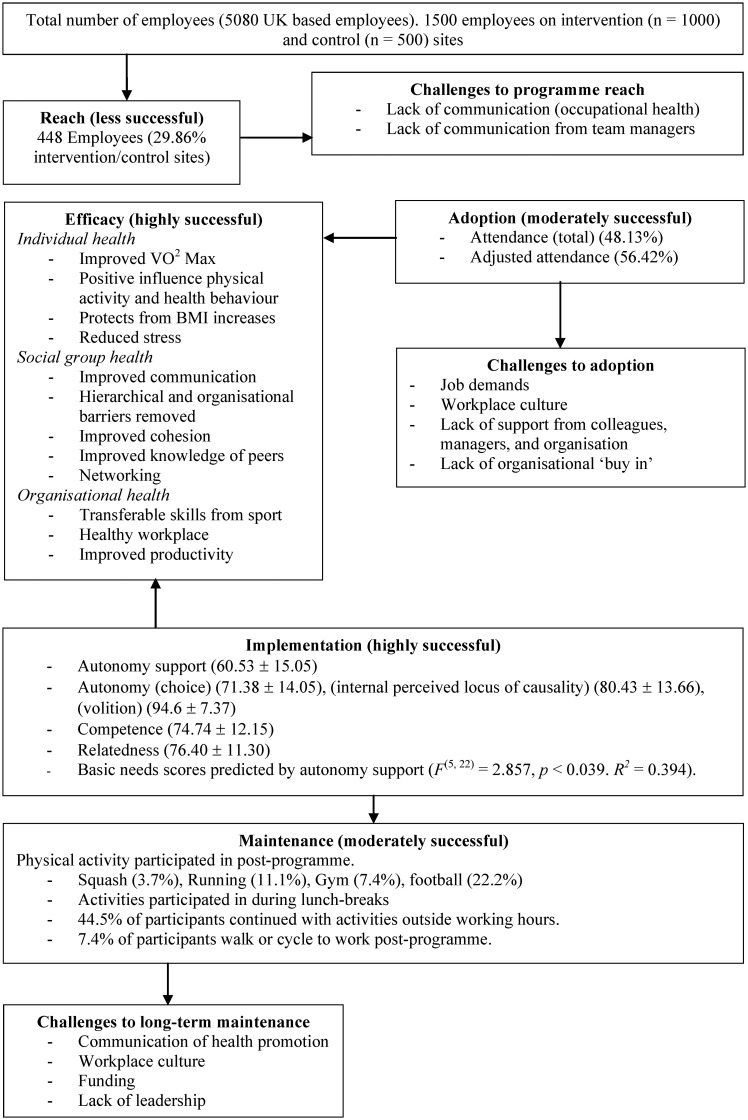
Schematic overview of findings.

RE-AIM dimensions (reach, efficacy, adoption, implementation, maintenance) were evaluated through triangulating the quantitative and qualitative data collected. Each dimension was rated on its applicability (i.e., the extent to which the collected data could accurately assess the dimension) and outcome (i.e., positive or negative outcome based on the data collected) (1 = *limited success*, 2 = *moderate success*, 3 = *highly successful*) [16]. A dimensional rating was determined by adding the applicability and outcome scores and then dividing by two. A schematic overview of the findings is provided in Figure 1.

## 3. Results

### 3.1. Reach (limited success)

The study was advertised to the workforce through emails, social media notifications (i.e., Yammer, internal intranet system), presentations to staff groups, and posters in locations with a high footfall (e.g., meeting spaces, social areas, cafes, toilets, lifts) for one month prior to the study commencing (May-June 2016). CTG reached 448 participants of the estimated 1500 employees working at the intervention and control worksites (29.86%). However, it appeared the programme had not been effectively communicated by the organisation or management teams to either the intervention or control worksites prior to the programme commencing:

‘People were asking, what are you doing, what's with this sport thing? How do you get involved in the sports challenge? There were a number of people who hadn't heard about it. The company could have helped sell it more. It wasn't sold very well around the workplace [Dominic, aged 47, male team manager, CTG participant].’

### 3.2. Efficacy (highly successful)

Intention-to-treat analysis using mixed-ANOVAs found CTG to significantly improve VO_2_ Max (*P* < 0.002), interpersonal communication within teams (*P* < 0.05) and mean weekly physical activity duration (*P* < 0.002) when compared to the control group [8]. Other individual, social-group and organisational health outcomes showed non-significant improvements in the intervention group. The full results of the study can be found in Brinkley et al [8].

### 3.3. Individual health

Employees described their participation in workplace team sports as positively influencing their perceptions of their own competence in physical activity and in their self-efficacy (confidence) in taking part in in physical activity outside the programme:

‘It is the sport and what that has enabled me to do since. I've now been going out for walks and doing more, whereas before I just used to sit in front of the TV or do the minimum. Sport gave me the confidence that I am able to do this. I have got the confidence to go out and enjoy myself and forget other people [Team manager, female aged 50, CTG participant].’

‘It gives you an intro to sport, it helped me take up sport in my spare time. I enjoyed it and because of this I have taken up cricket outside work where one of my mates plays for a cricket club on a regular basis [Advisor, male aged 41, CTG participant].’

Participation in CTG offered an activity which had the capacity to relieve personal and workplace stress. For example, one participant described how the programme offered respite and an activity to disassociate from personal and workplace stress and tension:

‘I am going through a lot of personal issues in my life. For me it was also a chance to get some tension relief by actually doing some physical exercise, some running around. It was a complete break from work, so turning off your brain from focusing on a PC screen and doing something that is completely different. It is a chance to totally forget about it, and do something that is releasing endorphins within the body, I don't know what ever sport does for you that makes you feel better, because I do feel better after playing sport. [IT analyst, male aged 44, CTG participant].’

### 3.4. Social group health

CTG was reported to improve interpersonal relationships. Participation provided an environment whereby colleagues and superiors could interact without the logistical (e.g., differing offices and departments) and hierarchical (e.g., differing levels of superiority) constraints present within the workplace. Participants described how this socially interactive environment promoted cohesion with their colleagues during and following participation:

‘The social bit was great. There were people who were there who were from different floors of the building, and I've never met them before. I might have seen them but I've never spoken to them. This actually broke down those barriers and yeah it was a good experience [Lead planning analyst, male aged 45, CTG participant].’

A cohesive environment appeared to have the capacity to influence interpersonal communication. Participants and champions described learning more about their colleagues' preferences, personal-life, personality and job role while participating in CTG. Frequently, a network within the organisation was identified as a result of an improved relationship:

‘I believe that it opens up channels of communication within the organisation that you wouldn't necessarily have or have been able to have used. So, in the past, if I had an issue I would have never normally have approached Sarah [CTG participant], I had no idea what she does, but now all of a sudden, I know which floor she is based, so if I have something I am not certain of I might go and hunt her down and ask her. I have a familiar face in that part of the organisation, which I wouldn't have had if I hadn't gone and played team sport [Team manager, male aged 47, CTG participant].’

In terms of productivity, some participants and champions effectively used the networks they had created during CTG within their role in the organisation:

‘So now I have a network of people including Gill in HR. She has helped me on numerous occasions, because I thought I know her, I will go and have a chat to her, and I did. There was also a guy, and we were competitive with each other. Tom, I have been to him on a number of occasions to help my team with a fix that needs fixing. So, I didn't know him before that, and I just thought it didn't matter what happened in the hall, as soon as we finished from there, we were back to being a normal selves and we didn't hold grudges, no matter what happened in the hall [Team manager, female aged 50, CTG participant].’

### 3.5. Organisational health

Adaptations in individual health and developments in group cohesion may have the capacity to improve productivity. Participants suggested the exertion associated with team sport improved concentration and focus:

‘Every Wednesday afternoon, I could really see a difference in my concentration, I stayed even later Wednesday evening purely because I had the energy to do that. It lifted the fog, because I was buzzing. I know there is a hormone that gets released but I don't know what that is, but that was it, it was really helping [Team manager, female aged 50, CTG participant].’

### 3.6. Adoption (moderately successful)

Adoption refers to the number of participants willing to participate in an intervention over time, and the reason behind their engagement or disengagement from the programme [7]. Twenty-seven out of 28 participants attended at least one CTG session (no participants attended all CTG sessions). Excluding the one participant who did not attend any intervention sessions, the average attendance across the 27 participants was 48.13%. The adjusted attendance rate for frequent nonattendance (<25% of intervention sessions) (*n* = 23 participants) was 56.42%.

**Figure 2.**
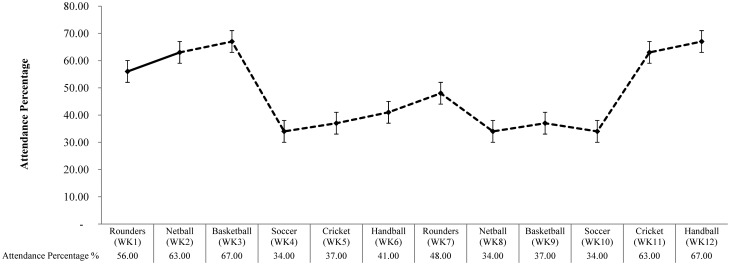
Attendance over 12-weeks (WK: Week; Standard error bars are displayed).

At least 75% of participants (21 of 28 participants) completed all T^1^ outcome measures across the study duration. A series of one-way ANOVAs (see Figure 2) examined week-by-week attendance. No significant differences in attendance rates were observed between weeks 1–9 and weeks 11–12 (*P* > 0.05). A significant difference between participants was observed at week 10 (*P* < 0.037, η^2^ = 0.276). Post-hoc tests revealed significantly less participants attended week 10 (soccer) compared to all other weeks (*P* < 0.037). Soccer was the least successful sport in weeks 4 and ten (34% attendance). Basketball in week 3 and handball in week 10 were the most successful sports with 67% attendance.

The attendance of participants was challenged by interpersonal and organisational factors. Despite sessions being conducted during lunch-breaks, participants described how the demands within the workplace challenged their regular participation:

‘Work gets in the way. There was always a meeting at eleven o'clock which was supposed to finish at twelve, but it often carried on till ten past, twenty past. I thought stuff it, and started walking down the stairs while they were talking to me and got changed downstairs and said look I've got to go now. I should have said I can't do this, because I had done my actions, I had my time to talk. But it is just not the done thing to disappear [Systems specialist, male aged 53, CTG participant].’

Despite CTG being supported by the board and senior management, several participants believed the organisation did not provide enough support for their attendance:

‘There is a lack of buy in from the organisation with things like this. I think with the business it comes down from like the board and think there is it well communicated, but when it is individual things like this, they [colleagues and superiors] haven't always done as well. I don't remember that much pushing or support from the organisation about it [Team manager, male aged 47, CTG participant].’

A lack of substantial support for CTG may be explained by the pressure placed on downsizing the organisation due to recent financial problems:

‘There is not enough people doing most of the jobs, they slimed it all down and restructured it. Everyone is so busy, so as soon as you throw extra responsibilities in the mix, they commit thinking it is only twelve weeks, it is only a short-term thing, and then actually that is a lot [Finance manager, female aged 31, CTG champion].’

### 3.7. Implementation (highly successful)

#### 3.7.1. Theoretical underpinnings

CTG was underpinned by Self-Determination Theory, which proposes that meeting basic needs for autonomy, competence and relatedness fosters optimal functioning, well-being and autonomous motivation [9],[10]. Thwarting these basic needs are known to lead to poor wellbeing and controlled forms of motivation [9],[10]. For example, providing sessions which do not offer participants a choice and ownership (autonomy), sports which are not adaptable or do not have transferable skills (competence), and an environment which is not socially supportive (relatedness). Our CTG intervention programme supported basic needs through an autonomy supportive leadership style [8]. See Brinkley et al. [8] for a comprehensive overview.

#### 3.7.2. Autonomy support (workplace champions)

A key component of CTG was the delivery of team sport. This was conducted by two female workplace champions. Prior to the programme commencing, both workplace champions completed training delivered by the first author (i.e., education sessions covering; autonomous motivation, supporting basic needs and leading intervention sessions). This encouraged workplace champions to adopt an autonomy supportive leadership style which welcomed the thoughts and perspectives of participants, provided knowledge to enable participation, and created a setting for self-directed participation. Working together, both workplace champions led intervention sessions using this autonomy supportive style:

‘I was making sure everybody was involved and people weren't getting left out and that people understood what they were going to have to do. With football for example, people assume that everybody knows what the rules are and how to kick a ball and stuff like that, and a lot of people don't. It's about making sure people know they are in a safe environment and you know if that's worked because they say actually look I don't get it Laura [Business analyst, female aged 36, CTG Champion].’

Subsequently, on the process evaluation questionnaire, high ratings of autonomy support (i.e., over the mid-point of 50), as measured through the Sport Climate Questionnaire short-form, were reported by CTG participants (mean 60.53 ± 15.05).

#### 3.7.3. Autonomy

Qualitative data reflects how workplace champions offered participants the autonomy to shape and contribute to the structure of the sessions:

‘She [workplace champion] offered us the choice on how we could solve different numbers on each team, we decided that we would let one person move up and down the pitch on one team in netball. It helps because sometimes someone is quite strong willed and strong minded, they will try and fit the sport around their best needs, so they can win. Having someone there independently helps to set up the sports, give you the rules, give you the equipment and off you go [Male systems specialist, male aged 53, CTG participant].’

Offering a choice of sports provided volition and a sense of ownership for participants. Participants explained how ownership and a choice created markers of autonomous motivation such as enjoyment and internalised-control:

‘We never had a set game, we changed the rules as we were going along. I think it worked well, it allowed us to own the rules. There is a real sense of ownership there. Changing the rules as we go along, that is reflective of how I really enjoyed the sessions. It felt like we owned it with a little bit of input or guidance from the champions [Project manager, male aged 39, CTG participant].’

Participants scored highly (mid-point 50) on markers of autonomy such as choice (71.38 ± 14.02), internal perceived locus of causality (80.43 ± 13.66) and volition (94.6 ± 7.37).

#### 3.7.4. Competence

Data from the process evaluation questionnaire found that CTG participants rated highly on basic needs for sports competence following participation in the programme (74.74 ± 12.15). The intervention programme was designed to support needs for competence by offering participants sports that could be adapted to their own rules, and which required skills and traditions which were transferable between the sports such as catching in cricket and rounders, and similar spatial awareness skills for soccer and handball [9],[10]. During interviews, participants described how the ease of learning sports through adaptions in their rules, traditions and style of play supported needs for competence:

‘Yeah they were all very easy to learn I think. You have got to appreciate that we are not there at the Olympics. So, do we have to follow every single rule, no, it is just a little bit of fun. So, in that, it makes it easy to learn because you just need to know the basics really [Senior advisor, female aged 25, CTG participant].’

Some participants engaging in CTG had experiences of playing team sport (e.g., football). For these participants, the novelty of some sports played during CTG provided a new skill to master, an intensity to participate at or tactic to exploit:

‘Handball, I really enjoyed that if I'm honest. It was something completely different, it was something I've never really tried before. To try something different, and to take to it straight away, that was really nice. One of the things I liked about it. It's a simple game to play. I think it might be because it is high intensity. I think the high intensity could be the thing there, because you are really moving, you are really pushing yourself [Lead planning analyst, male aged 45, CTG participant].’

Furthermore, the reports of participants indicate participation in CTG offered an environment which supported needs for competence. Social support and observing changes in personal skill, fitness and technical proficiency appeared to foster needs for competence, optimal functioning and wellbeing:

‘Day one, I had to stop a couple of times, but I would have thought I would have had to stop a lot more, and the team were great, you felt safe in that environment, nobody was kind of like judging, which is what I am use too. The champions have the patience, and some of the guys would help and say don't do it that way, do it this way, and I think that helped, particularly with football, because I was ok when I was walking, but as soon as I had to run and had to lift my foot up and the ball came at speed, it just went underneath my foot. That sort of gave me the confidence that I can do it, I am still able to pick up stuff that I learnt when I was eleven or twelve. They want you to succeed, and I think that is what maybe rubbed off, so I haven't fell over, I've held my own, I've done whatever I need to do and I felt on top of the world if you like [Team manager, female aged 50, CTG participant].’

#### 3.7.5. Relatedness

Relatedness is feeling valued, supported and understood by a social group (e.g., colleagues, workplace team). Relatedness was rated highly by CTG participants in the process evaluation questionnaire (mean 76.40 ± 11.30). Supported needs for relatedness may be explained by the qualitative data collected. The CTG intervention offered a form of physical activity where colleagues shared the organisational challenges to physical activity within the workplace:

‘We all understand the pressures we are under, we all understand why we are doing it [CTG], I think because the company approves it, it all helps, because you all work under the same flow processes, you all work in a similar way [Systems specialist, male aged 53, CTG participant].’

This sense of group identify was also described by participants to promote group cohesion and provide encouragement to participate:

‘I found it really encouraging playing with these people. Because they are there for the same reason as you, they want to get a bit fitter, so we are there to encourage not to criticize, people are always different skill levels to everybody else, so it's just about encouraging them and playing well together [Technical expert, male aged 50, CTG participant].’

Furthermore, participants explained that the cohesive group environment associated with CTG could provide social support and promote self-efficacy to participate:

‘I am big lady and because I am unfit, I am fifty, I thought people would judge and say I don't want her on my team and that's why I did say to a few people I knew, don't pick me last, and I am not that kind of person normally, but that was going back to school, where it was a little bit like that, but I didn't feel any of that here. I felt as if, whatever team I was on, I gave it my all, I couldn't do anymore, and I encouraged other people and got encouragement from other people. They wanted me to succeed and the team to succeed, rather than looking after themselves [Team manager, female aged 50, CTG participant].’

#### 3.7.6. Are basic needs predicted by an autonomy supportive workplace team sport programme?

Descriptive statistics and Pearson correlations were computed between Sport Climate Questionnaire short-form and Basic Needs in Sport Scale scores. Autonomy support was significantly correlated with competence (*R* = 0.354, *P* < 0.032), internal perceptions of the locus of causality (*R* = 0.251, *P* < 0.014), volition (*R* = 0.568, *P* < 0.001) and relatedness (*R* = 0.420, *P* < 0.013). Competence was significantly correlated with choice (*R* = 0.839, *P* < 0.0001), internal perceptions of the locus of causality (*R* = 0.802, *P* < 0.0001), volition (*R* = 0.527, *P* < 0.002) and relatedness (*R* = 0.416, *P* < 0.014). Perceptions of choice were significantly correlated with internal perceptions of the locus of causality (*R* = 0.641, *P* < 0.0001) and volition (*R* = 0.494, *P* < 0.004). Internal perceptions of the locus of causality were significantly correlated with volition (*R* = 0.579, *P* < 0.001) and relatedness (*R* = 0.574, *P* < 0.001). Volition was significant correlated with relatedness (*R* = 0.331, *P* < 0.043). AS and relatedness were not correlated with choice. Standard multiple regression was conducted with autonomy support and basic needs (i.e., competence, choice, internal perceptions of the locus of causality, volition and relatedness). Basic needs scores were significantly predicted by autonomy support (*F*^(5, 22)^ = 2.857, *P* < 0.039. *R^2^* = 0.394). This analysis was sufficiently powered (*1-β* = 0.845).

### 3.8. Maintenance (moderately successful)

Thirteen participants (44.5%) continued with the leisure-time and workplace physical activity they were participating in prior to CTG. Fourteen participants participated in additional physical activity in their workplace or with their colleagues since completing the CTG intervention programme. More specifically, since completing CTG two participants had begun active commuting (7.4%), and during the working week, two participants attended the gym at lunch (7.4%), six participants had taken up indoor football during lunch (22.2%), one participant had taken up squash during lunch (3.7%) and three participants has started running during lunch (11.1%).

However, many participants identified challenges in maintaining participation in team sport for the long-term. These included communication, funding and leadership within the organisation. Health and wellbeing messages and programmes appear to have a low reach due to the style and level of communication adopted by the organisation. Participants described the importance of changing the culture within the workplace. Despite pressure from the employer to implement novel forms of workplace health promotion, a culture driven by health and safety and a work-and high work demands challenged the maintenance of CTG:

‘I think quite often, if people are out there enjoying it then other people want to get involved as well and it kind of has that effect. I think there needs to be something or someone higher up that says there be no meeting booked between this time and this time, because people don't have the time. I think we would struggle to change the way it is at the minute [Senior advisor, female aged 25, CTG participant].’

CTG was funded by the researchers' university, therefore eliminating pressure placed on funding participation. However, when the long-term maintenance of the programme was discussed challenges relating to self-funding participation were highlighted:

‘I think if people had funded it themselves, you may have had less interest, I think funding it gives people the chance to try it [Lead planning analyst, male aged 45, CTG participant].’

Several participants commented on how their employer could use company resources:

‘It's a multi-billion-pound company, we are talking about half a dozen footballs, a couple of cricket sets, we are talking about something that is significantly less outlet finically than my whole departments fund for the Christmas party [Advisor, male aged 41, CTG participant].’

For the maintenance of CTG to be a success, participants frequently commented upon the importance of a leader within the workplace to organise and deliver sport to colleagues:

‘I really hope we are able to carry it on, that's all I would like to say, and I would like for it to happen within our organisation and it to be supported by them, for them to find a champion and if they need someone to step up I think you could choose any one of the people you saw there and I think all of us could encourage more participation [Team manager, male aged 47, CTG participant].’

However, job demands and pressure from managers, challenged the potential of some participation championing CTG over the long-term:

‘I just wish we could continue doing it. But if we haven't got somebody leading it, it won't happen. I have not got enough time at the minute to lead it. My boss has got me running ragged [Technical expert, male aged 50, CTG participant].’

## 4. Discussion

This study reports the findings from our process evaluation of CTG [8], a team sport intervention programme designed for the workplace. Strength of our process evaluation is the use of triangulation, whereby data from several sources were evaluated together. We evaluated CTG through the RE-AIM framework. The *reach* of CTG had limited success. The *adoption* and *maintenance* were moderately successful at an individual level due to targeting and supporting autonomous forms of motivation in the intervention group. However, success at an organisational level was limited due to the work demands, and the culture of the organisation. CTG was highly successful in terms of its *efficacy* and *implementation*. The intervention programme offered novel and adaptable sports with transferable skills across the different sports through the leadership of workplace champions who provided a social environment that supported autonomy. This social component of the intervention was therefore effective in supporting participation in CTG.

CTG reached a modest percentage (29.86%) of the intervention and control worksites. Previously, the reach of workplace team sport studies has been poorly reported [5]. However, one intervention study reported a reach of 19.25%, albeit from 1000 employees [17]. In both cases, it is interesting that the reach of team sport is less than other forms of group physical activity such as activity challenges (32.24%) [18].

Reach was conservatively calculated from the two worksites (intervention and control) only. Whereas the 5080 employees represents the UK workforce and employees working remotely (to whom the programme was not communicated to), if implemented across the entire workforce, the actual reach of the programme may have been higher than what was reported here.

In the case of CTG, the reach of the programme may have been influenced by the communication strategy and management teams within the organisation. The 2016 annual report from the participating organisation consistently echoes the importance of participation in physical activity in the workplace^2^. However, the recruitment communication for CTG was reported as being ineffective by the participants in the process evaluation. One explanation offered by participants was a lack of support by middle management and team leaders. Whilst not reported as directly unsupportive, it appeared managers may have chosen to not raise their colleagues' awareness of the programme or to offer their direct approval for participation. Evidence shows that managers and superiors are influential stakeholders in encouraging participation and adherence to research studies and health promotion programmes [15],[19],[20]. In the case of CTG, participants drew negative social comparisons with the behaviour of their superiors. It is plausible these comparisons may have thwarted participant's needs for relatedness (e.g., to feel social supported) and therefore reduced participants' motivation for participation in CTG [21].

An alternative postulation to a low reach may be the perceived and actual demands placed upon employees within the workplace. Consistent with other FTSE 100 service organisations, employees in the current study were subjected to long working hours, in a culture where working non-stop is encouraged [20]. Job demands have also been found to also challenge a workplace walking programme [20]. If the reach of health promotion programmes (e.g., team sport), within workplace settings are to match their apparent efficacy, these challenges must be better addressed. For example, by creating a workplace culture that supports and encourages health promotion participation through work practises that promote flexible working [4],[15],[20].

The adoption of CTG can be considered moderately successful. Evidence shows workplace physical activity programmes typically have a low level of adoption over the short term [21],[22]. CTG was successful at engaging 75% of participants from T^0^ to T^1^. This figure is consistent with previous workplace team sport interventions at 12-weeks (75%) and at 40-weeks (58.5%) [23],[24].

Soccer was found to be the least successful sport in terms of attendance (34% in weeks 4 and 10), whilst basketball in week 3 and handball in week 12 proved to be the most popular with 67% attendance respectively. A low attendance rate for soccer may be explained by a lack of expertise in this sport. Sports such as soccer are known to challenge perceptions of competence, and likewise reduce participation [25]. However, given the limited number of sports investigated so far within the research, it may be unwise to conclude on the success and failure of certain sports within a workplace setting [5],[15]. Future workplace team sports intervention studies should to continue to explore the acceptability and feasibility, and tailor a range team sports into a workplace setting.

Modest attendance rates observed in CTG may be explained by several interpersonal, organisational and environmental factors [15]. The data reported within the current study indicates CTG participants were challenged by pressure to work during structured breaks despite the support of the organisation. Further, the culture within the workplace presents challenges whereby non-participants were willing to work through breaks and therefore implicitly pressure the attendance of their participating colleagues.

A further challenge faced by participants was the support of their organisation and more specifically, of their employer. Whilst messages of support are conveyed within annual reports and communications within the workplace, these appear not be consistent with employee attitudes to health promotion within the workplace. Past research exploring employers has drawn similar conclusions [26]. For example, despite acknowledging the importance of an active workforce, employers have been found to be resistant, uncertain and cynical to providing the support for workplace physical activity promotion [26]. In the case of CTG, downsizing within the organisation may have placed further stress on the workforce, and influenced the creation of cultures which support working non-stop.

Evidence indicates CTG is a programme with a high level of efficacy due to its successful implementation which was underpinned by Self-Determination Theory [9],[10], whereby needs for autonomy, competence and relatedness were supported. The research investigating workplace team sport has broadly been of low-quality [5]. The findings of the current evaluation provide a stronger case for employers considering promoting team sport. The current study indicates participation in workplace team sport may have a positive influence upon individual, social group and organisational health. This data is consistent with what has been reported previously [5],[15]. Though several benefits were highlighted which have not been identified by research, these include positive changes in behaviour and productivity [5].

At the end of the intervention, perceptions of needs the provision of autonomy support were rated as high (i.e., over the mid-point). Confirming the theoretical underpinnings of the programme, participants' perceptions of an autonomy supportive intervention predicted the satisfaction of basic needs. The findings of the current study also provide support for the recommendations of research, which suggests adult sports programmes should be underpinned and led by leaders (e.g., champions) supportive of basic needs [27].

Consistent with previous evidence examining an autonomy-supportive environment upon adults' sports participation and basic needs, autonomy support positively predicted autonomy (volition) [28],[29]. Qualitative data suggests intervention components such as promoting enjoyment and personal development in sport supports needs for autonomy. Evidence has linked these factors to intrinsic regulation (autonomous motivation), and controlled forms of motivation such as identified regulation [9],[10]. Given workplace champions adopted an autonomy supportive leadership style (e.g., offering choice and ownership) when delivering sports to their colleagues, it is theoretical logical participation in an intervention offering healthy motivation and functioning during sport would foster perceptions of autonomous participation [9],[10].

Our regression analyses found that autonomy support did not predict perceptions of competence [30]. However, our qualitative data suggests that key intervention components such as a taster session, adapting sport rules, providing novel sport, and promoting ‘success’ as personal development, rather than outright competition between peers foster needs for competence. Sports which are adaptable and use transferable skills should be considered within future programmes. Given the low statistical power achieved within our regression analysis, future studies should consider larger samples sizes when examining the impact of an autonomy supportive team sport programme on basic needs.

CTG was designed to support relatedness through the delivery of sessions by workplace champions, and employees participated with their colleagues and superiors. Following the completion of the intervention, autonomy support did not predict perceptions of relatedness. Our qualitative data however, provides support for this intervention component, in that participants sought relatedness (e.g., social support, empathy, cohesion, group identity) from their colleagues, superiors and employer to support participation. Promoting an autonomy supportive team sport with colleagues and through workplace champions may form a pragmatic method to support relatedness for future research.

If the benefits of workplace team sport are to be sustained, the maintenance of programmes must be improved. Despite participants adhering to physical activity (i.e., sport, active transport, exercise) post-programme, the maintenance of CTG can be considered moderately successful. In the case of CTG, several cultural challenges to long-term participation such as communication, funding and leadership of sport were identified. Workplace culture is known to influence participation in physical activity [15],[31]. A culture supportive of physical activity is understanding of flexible working and provides the necessary emotional, informational and tangible support for participation [15],[32]. In contrast, a culture not supportive of workplace physical activity promotes non-stop working and provides little ‘actual’ support for participation. In the case of CTG, a culture was identified which while offering messages of support through organisational outlets (i.e., message boards, reports, company communication), provided little support for employees participating in the programme or wishing to continue participation post-programme. These obstacles to participation are consistent with previous research [15].

If workplace team sports programmes such as CTG are to become a successful form of health promotion, a culture shift is required within workplaces. Evidence has indicated multicomponent interventions whereby theories of behaviour change are incorporated alongside organisational changes such as workplace culture may be more successful than behaviour change alone [31]. Although, CTG did adopt a participatory approach to account for the likeness of organisational challenges such as an unsupportive workplace culture, more could be done to affect these challenges occurring prior to the intervention commencing.

## 5. Conclusion

Through a mixed methods RE-AIM framework, the current study evaluated CTG, a team sport programme implemented within a FTSE 100 organisation. CTG was highly successful in terms of efficacy and implementation, the reach, adoption and maintenance could be improved. Changing the culture within organisations prior to interventions may better assist the reach, adoption and maintenance of future programmes.

## Footnotes

1 The first author AB is white British male aged 27 with experiences managing within a retail organisation. AB presented himself as a researcher willing to learn from the experiences of participants. AB is a competitive sportsman outside of academia (cycling). AB had no conflict of interest or investment in the participating organisation.

2 Distinguishable data (e.g., direct quotes/data) from annual reports not presented to protect the identity of the participating organisation, and employer.
